# Supplementary figures and images for: Elevation of MPF and MAPK gene expression, GSH content and mitochondrial distribution quality induced by melatonin promotes porcine oocyte maturation and development in vitro
Source: PeerJ. 2020 Oct 5;8:e9913. doi: 10.7717/peerj.9913 (PMC7543723; doi:10.7717/peerj.9913)

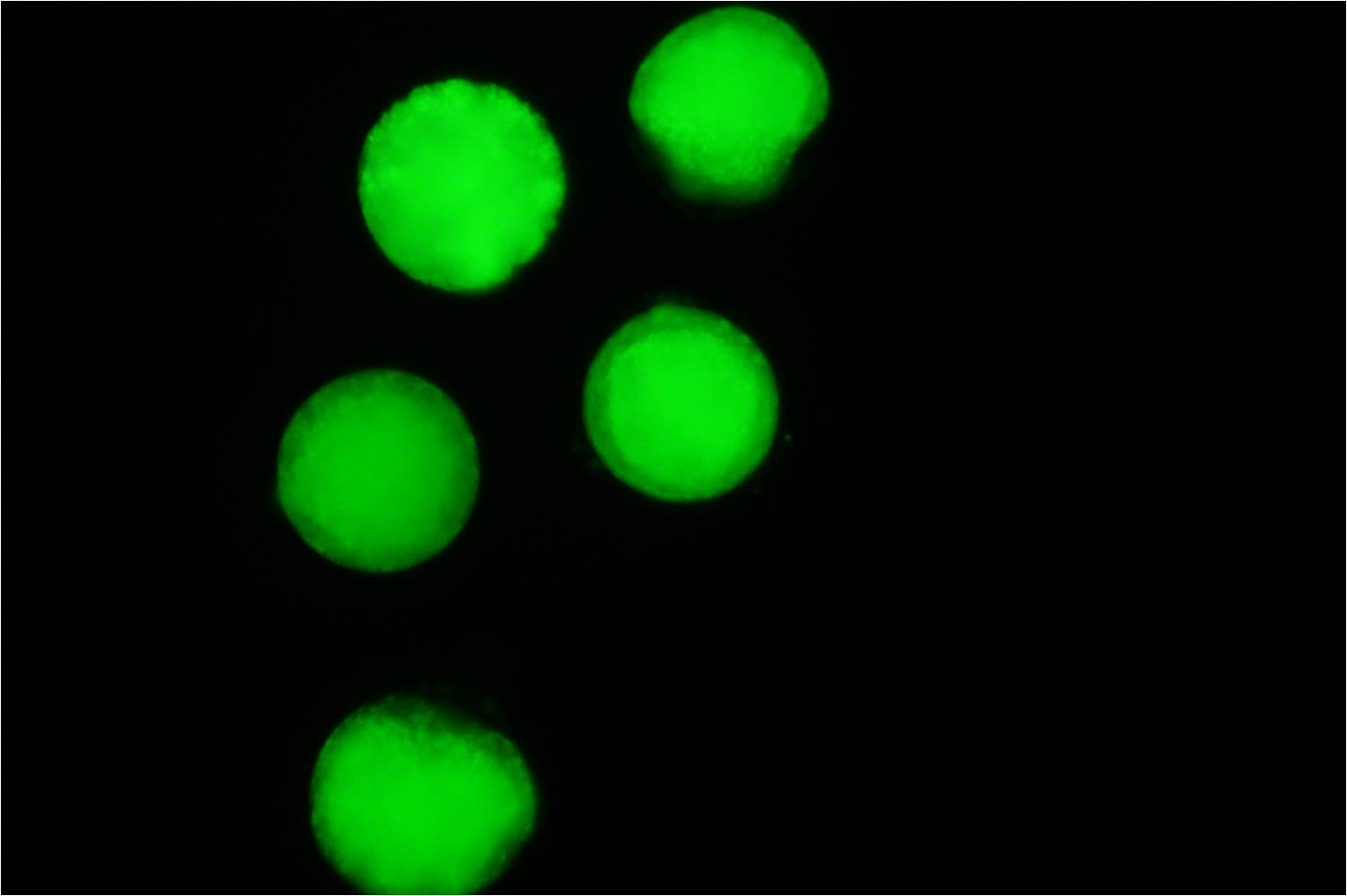

Supplement: Supplemental Information 2 — A: Stereomicroscopic examination of oocytes. B: FDA staining validated oocyte survival. These oocytes exhibited fluorescence in the ooplasmic membrane. C: Dead oocytes showed no or very low fluorescence. D: The stereoscopic examination of the matured oocytes with the first polar body expulsed. E: The matured oocytes were stained with fluorescent dye Hoechst 33342, where both the polar body and the nucleus exhibited fluorescence. F: Oocytes with no polar body expulsed were also stained with fluorescent dye Hoechst 33342, where only the nucleus exhibited fluorescence. [file peerj-08-9913-s002.zip › B, survival oocytes.jpg]

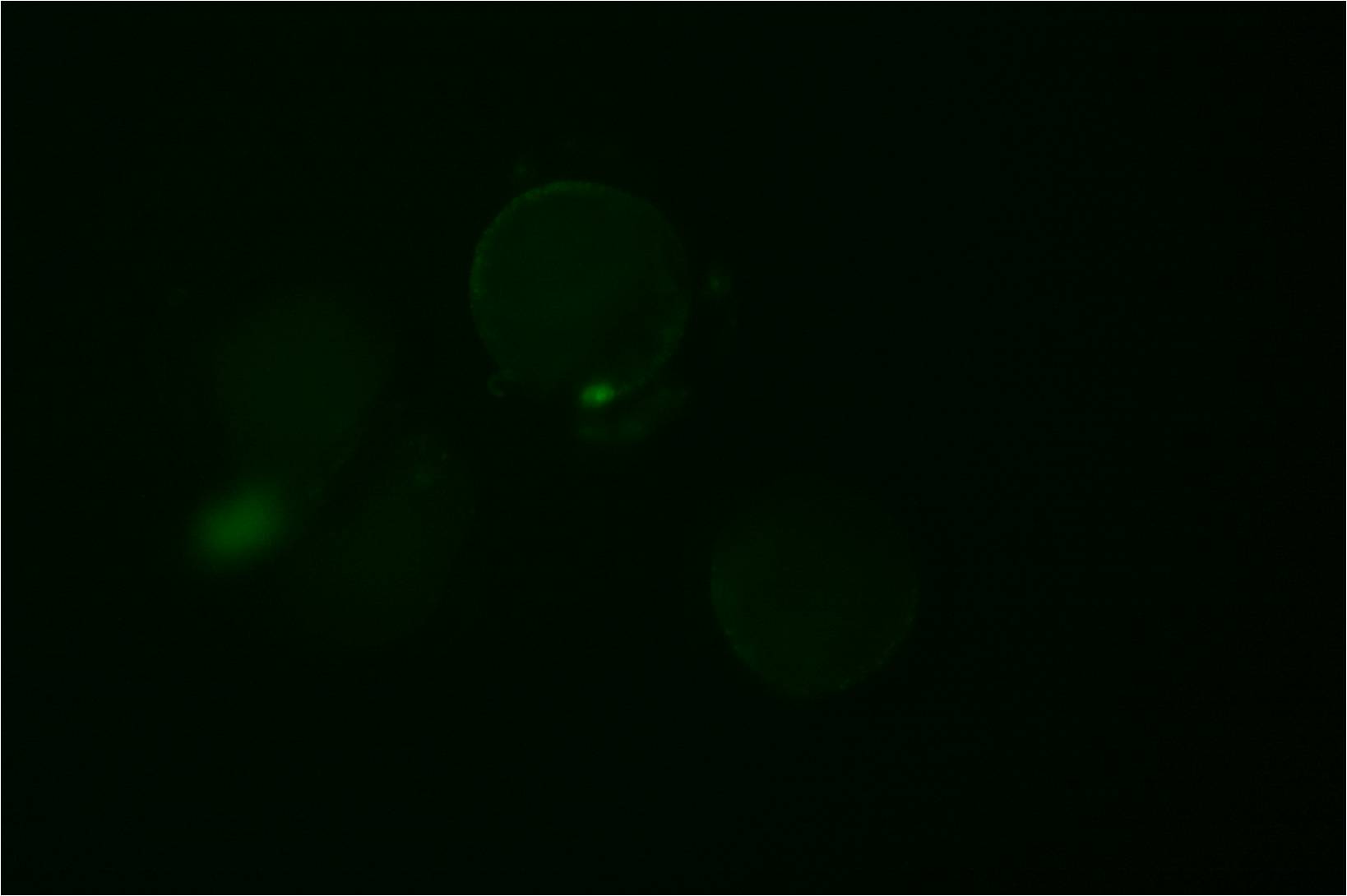

Supplement: Supplemental Information 2 — A: Stereomicroscopic examination of oocytes. B: FDA staining validated oocyte survival. These oocytes exhibited fluorescence in the ooplasmic membrane. C: Dead oocytes showed no or very low fluorescence. D: The stereoscopic examination of the matured oocytes with the first polar body expulsed. E: The matured oocytes were stained with fluorescent dye Hoechst 33342, where both the polar body and the nucleus exhibited fluorescence. F: Oocytes with no polar body expulsed were also stained with fluorescent dye Hoechst 33342, where only the nucleus exhibited fluorescence. [file peerj-08-9913-s002.zip › C, dead oocytes.jpg]

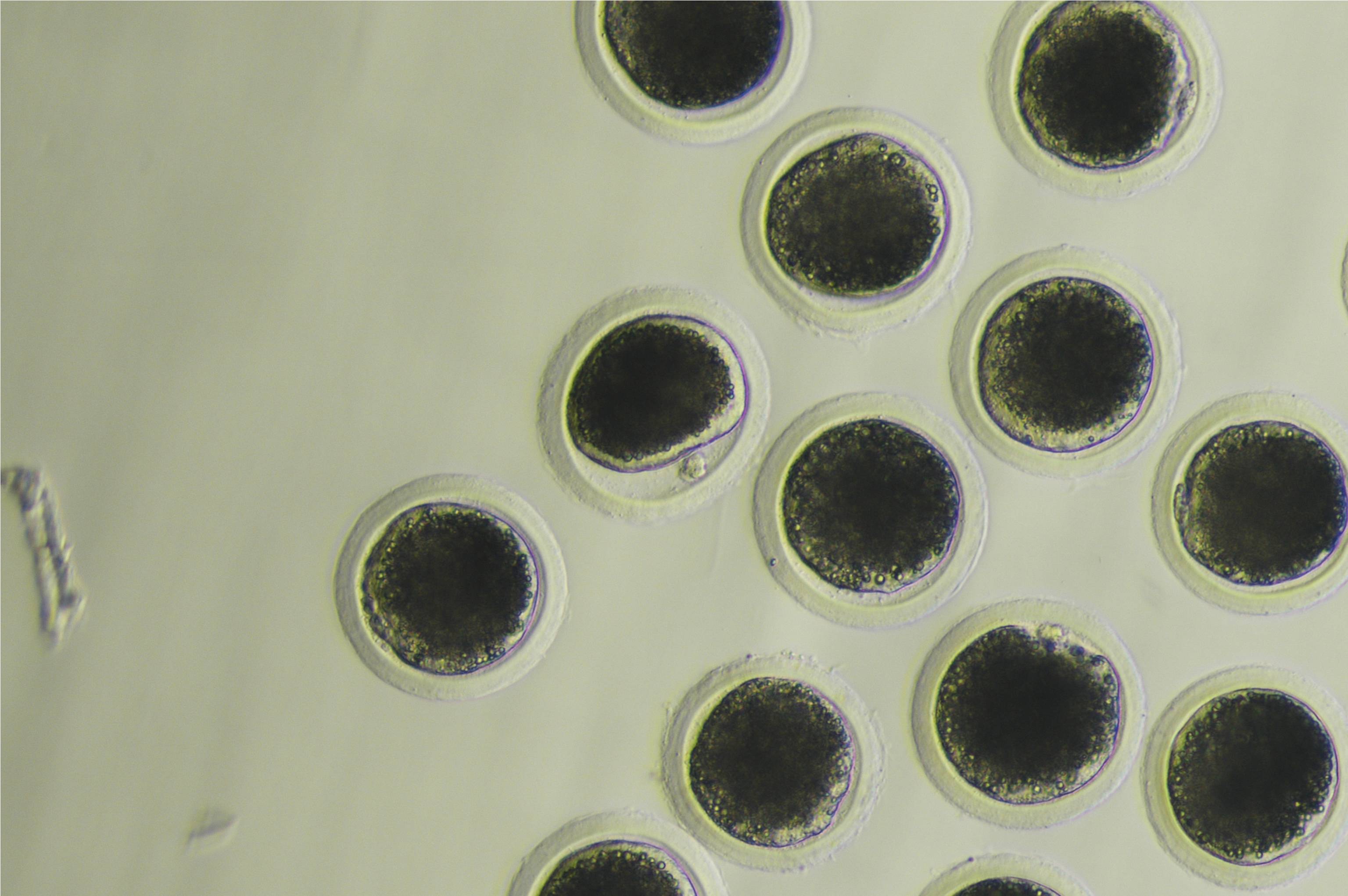

Supplement: Supplemental Information 2 — A: Stereomicroscopic examination of oocytes. B: FDA staining validated oocyte survival. These oocytes exhibited fluorescence in the ooplasmic membrane. C: Dead oocytes showed no or very low fluorescence. D: The stereoscopic examination of the matured oocytes with the first polar body expulsed. E: The matured oocytes were stained with fluorescent dye Hoechst 33342, where both the polar body and the nucleus exhibited fluorescence. F: Oocytes with no polar body expulsed were also stained with fluorescent dye Hoechst 33342, where only the nucleus exhibited fluorescence. [file peerj-08-9913-s002.zip › D,first polar body under stereomicroscopy.jpg]

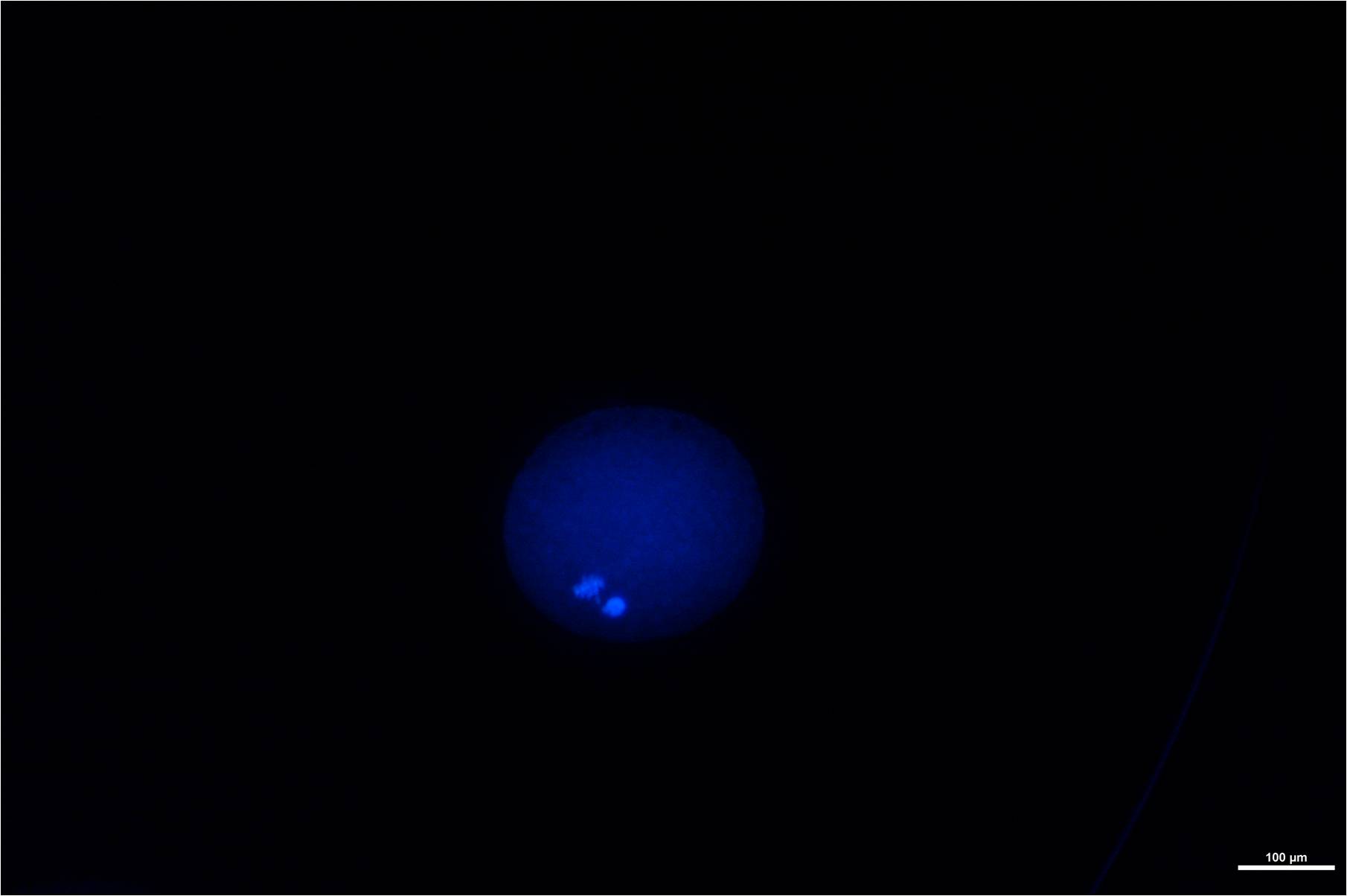

Supplement: Supplemental Information 2 — A: Stereomicroscopic examination of oocytes. B: FDA staining validated oocyte survival. These oocytes exhibited fluorescence in the ooplasmic membrane. C: Dead oocytes showed no or very low fluorescence. D: The stereoscopic examination of the matured oocytes with the first polar body expulsed. E: The matured oocytes were stained with fluorescent dye Hoechst 33342, where both the polar body and the nucleus exhibited fluorescence. F: Oocytes with no polar body expulsed were also stained with fluorescent dye Hoechst 33342, where only the nucleus exhibited fluorescence. [file peerj-08-9913-s002.zip › E,first polar body expulsed.jpg]

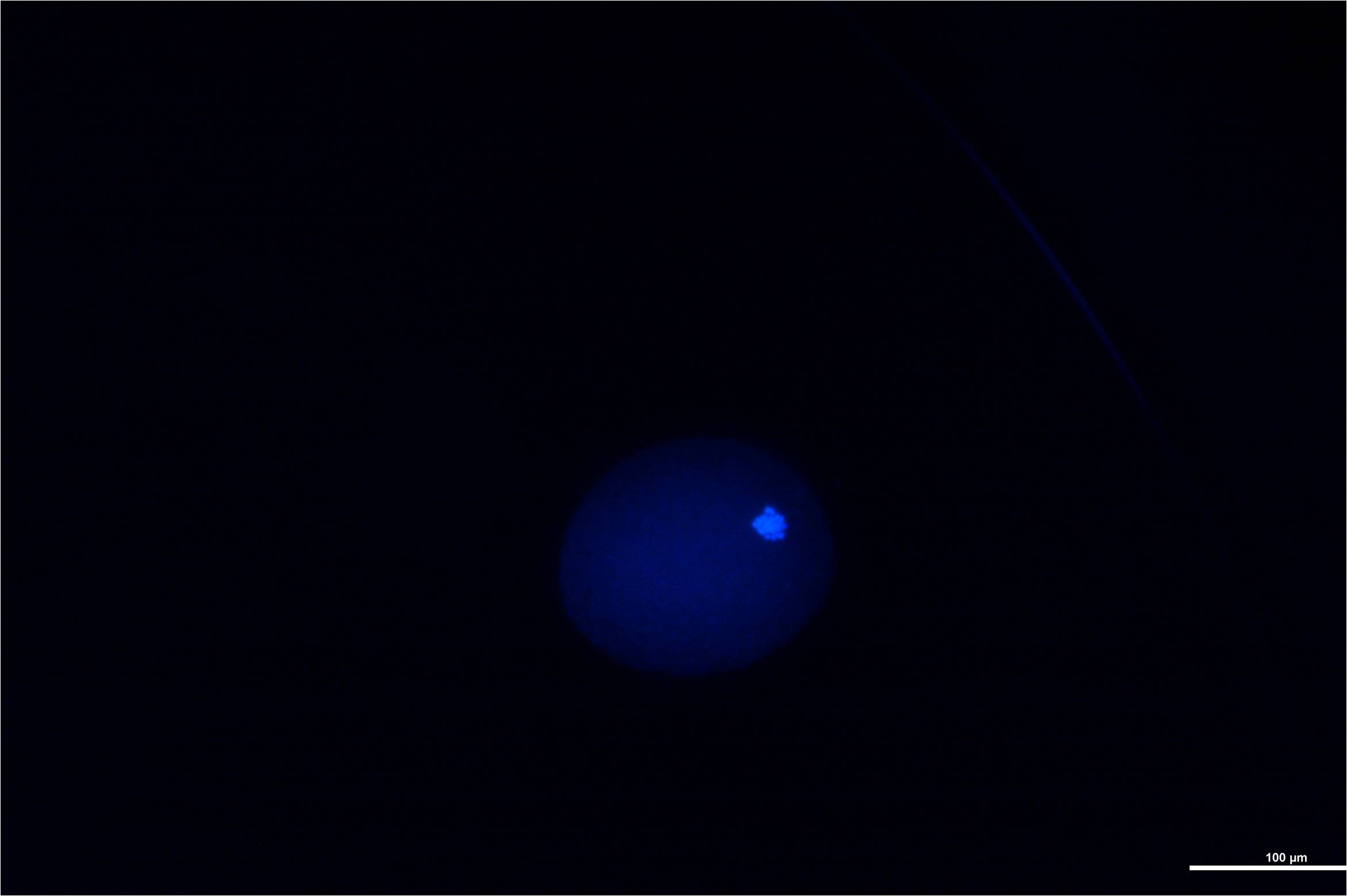

Supplement: Supplemental Information 2 — A: Stereomicroscopic examination of oocytes. B: FDA staining validated oocyte survival. These oocytes exhibited fluorescence in the ooplasmic membrane. C: Dead oocytes showed no or very low fluorescence. D: The stereoscopic examination of the matured oocytes with the first polar body expulsed. E: The matured oocytes were stained with fluorescent dye Hoechst 33342, where both the polar body and the nucleus exhibited fluorescence. F: Oocytes with no polar body expulsed were also stained with fluorescent dye Hoechst 33342, where only the nucleus exhibited fluorescence. [file peerj-08-9913-s002.zip › F,no first polar body.jpg]

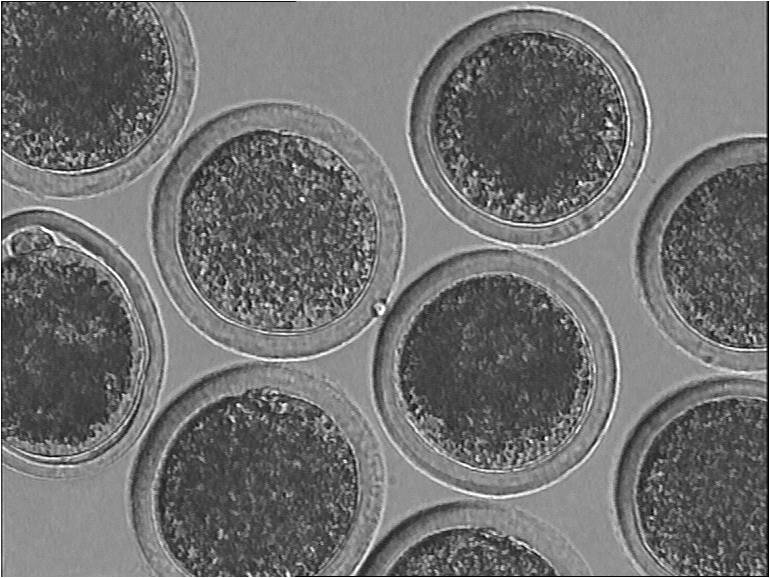

Supplement: Supplemental Information 2 — A: Stereomicroscopic examination of oocytes. B: FDA staining validated oocyte survival. These oocytes exhibited fluorescence in the ooplasmic membrane. C: Dead oocytes showed no or very low fluorescence. D: The stereoscopic examination of the matured oocytes with the first polar body expulsed. E: The matured oocytes were stained with fluorescent dye Hoechst 33342, where both the polar body and the nucleus exhibited fluorescence. F: Oocytes with no polar body expulsed were also stained with fluorescent dye Hoechst 33342, where only the nucleus exhibited fluorescence. [file peerj-08-9913-s002.zip › A, stereomicroscopic examination of oocytes.jpg]

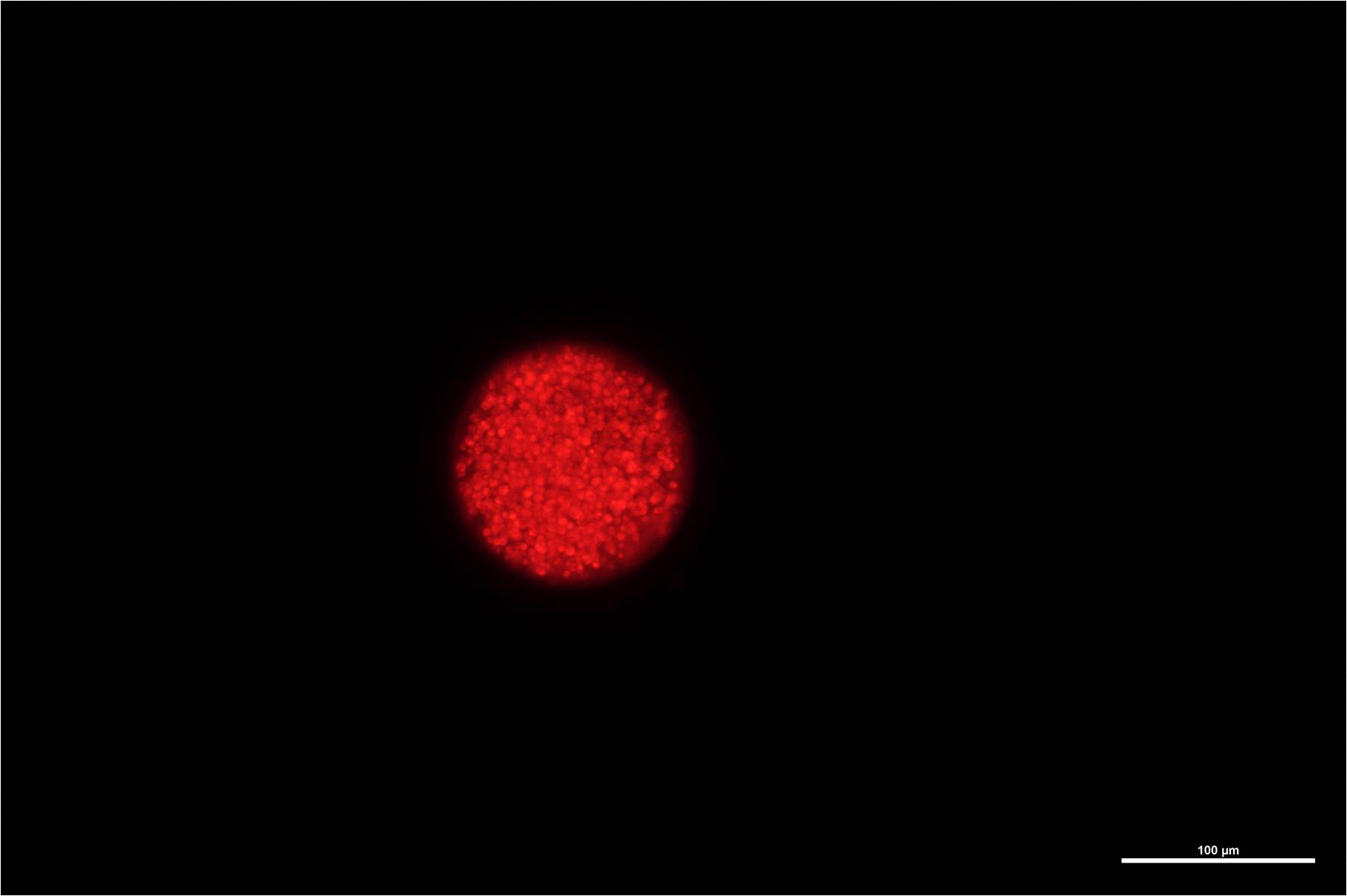

Supplement: Supplemental Information 3 — A: mitochondrial better distribution. B-E: mitochondrial heterogeneous and uneven distribution. [file peerj-08-9913-s003.zip › B heterogeneous and uneven distribution.jpg]

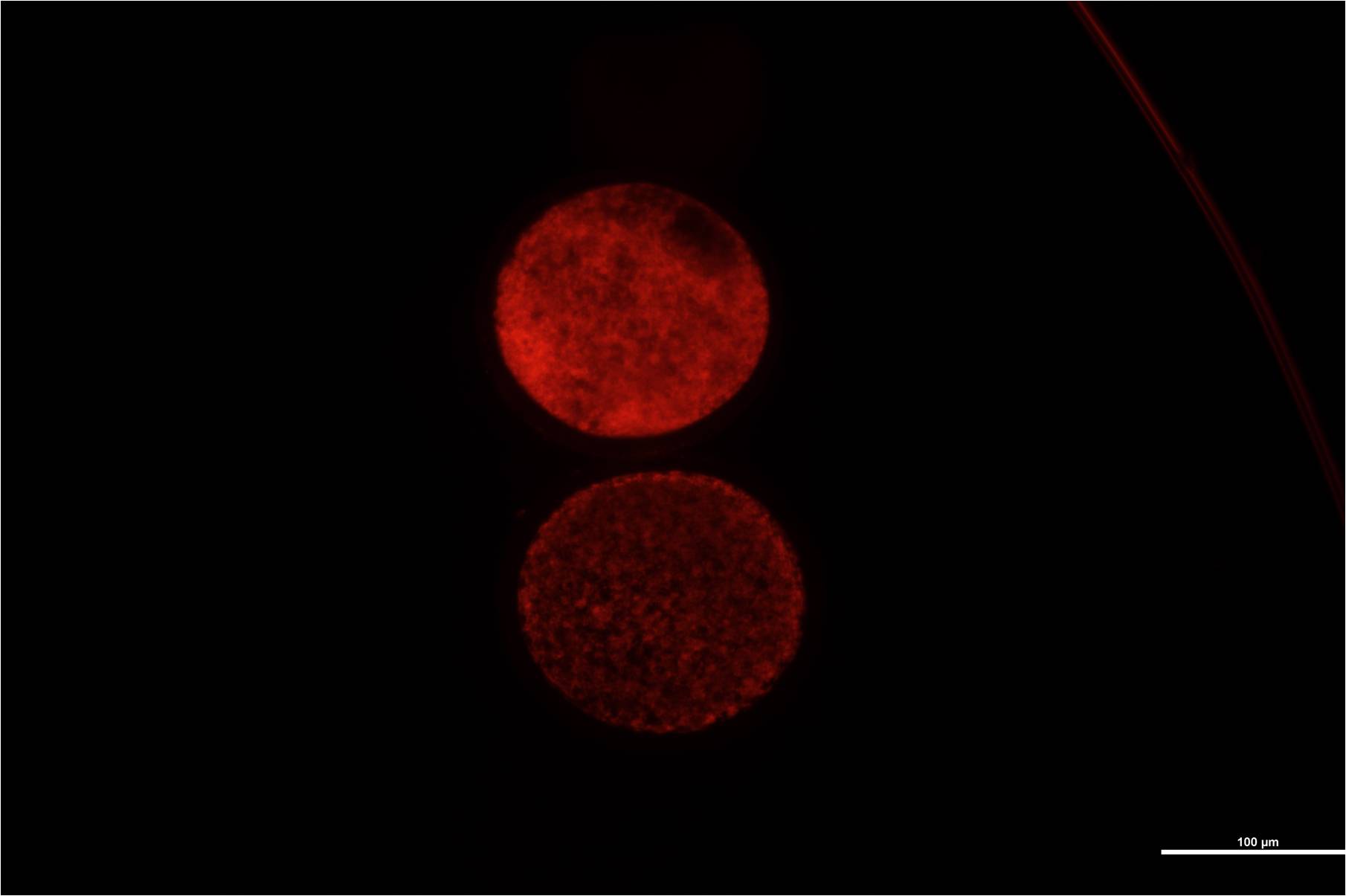

Supplement: Supplemental Information 3 — A: mitochondrial better distribution. B-E: mitochondrial heterogeneous and uneven distribution. [file peerj-08-9913-s003.zip › C heterogeneous and uneven distribution.jpg]

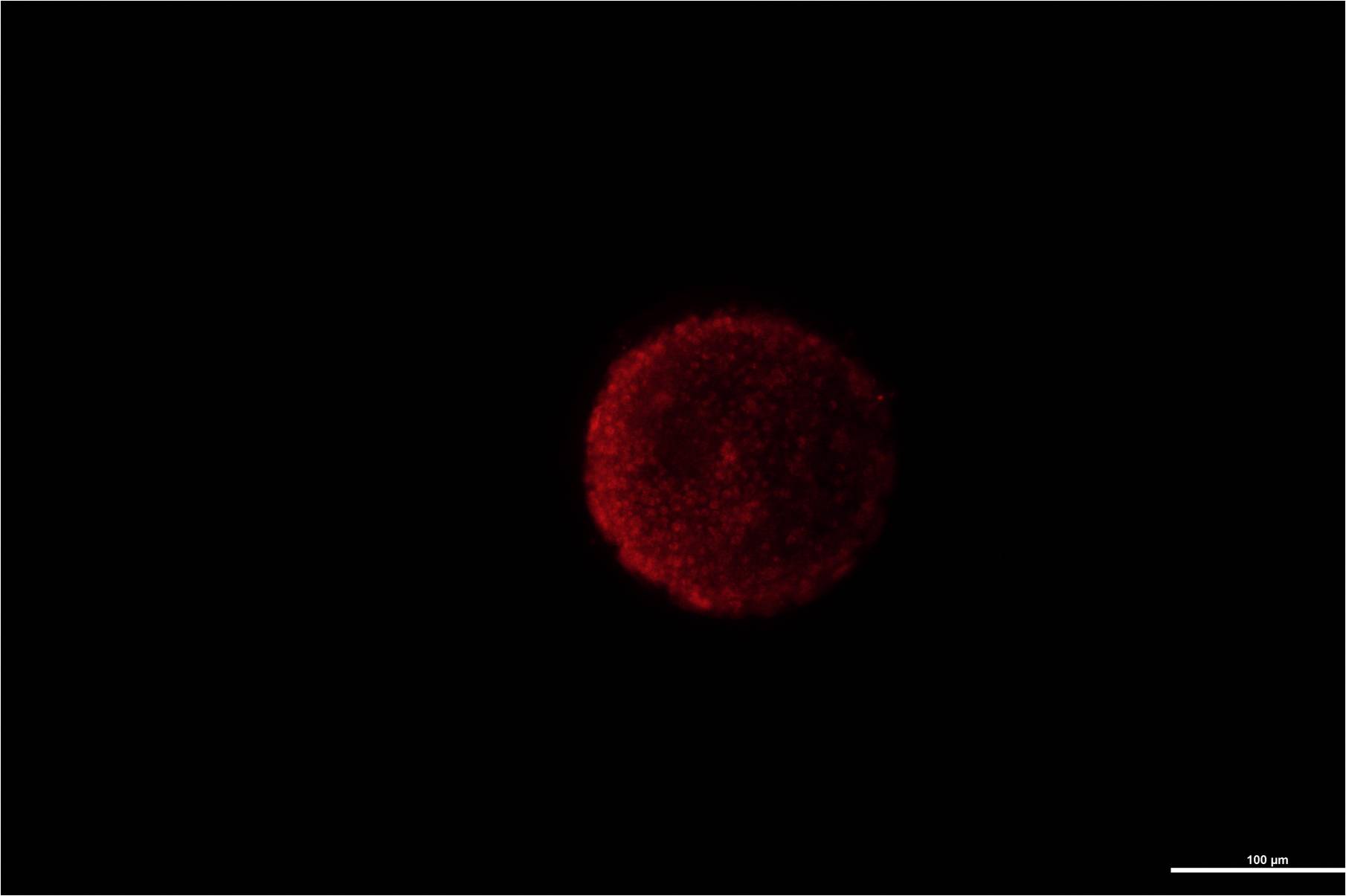

Supplement: Supplemental Information 3 — A: mitochondrial better distribution. B-E: mitochondrial heterogeneous and uneven distribution. [file peerj-08-9913-s003.zip › D heterogeneous and uneven distribution.jpg]

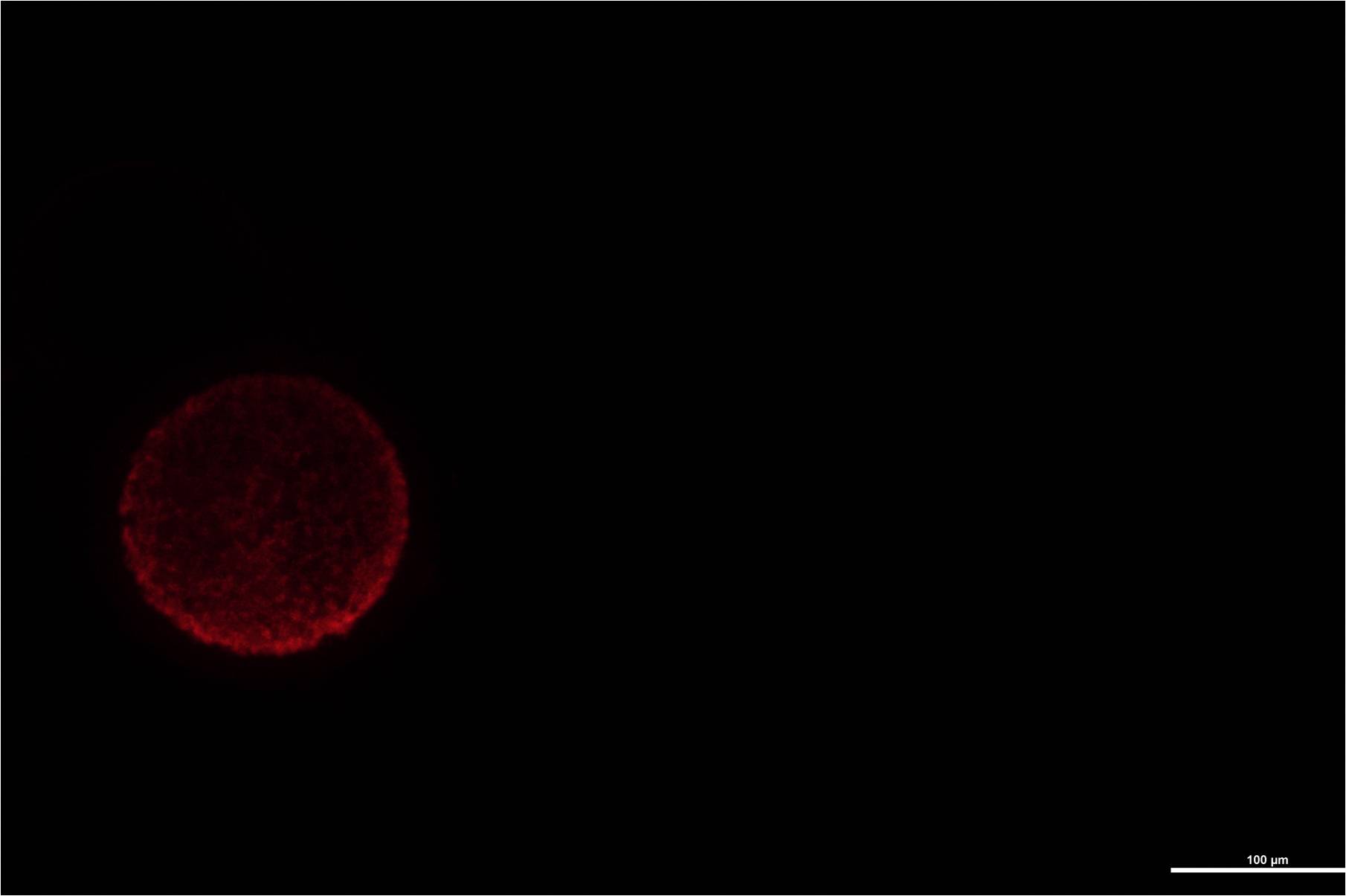

Supplement: Supplemental Information 3 — A: mitochondrial better distribution. B-E: mitochondrial heterogeneous and uneven distribution. [file peerj-08-9913-s003.zip › E heterogeneous and uneven distribution.jpg]

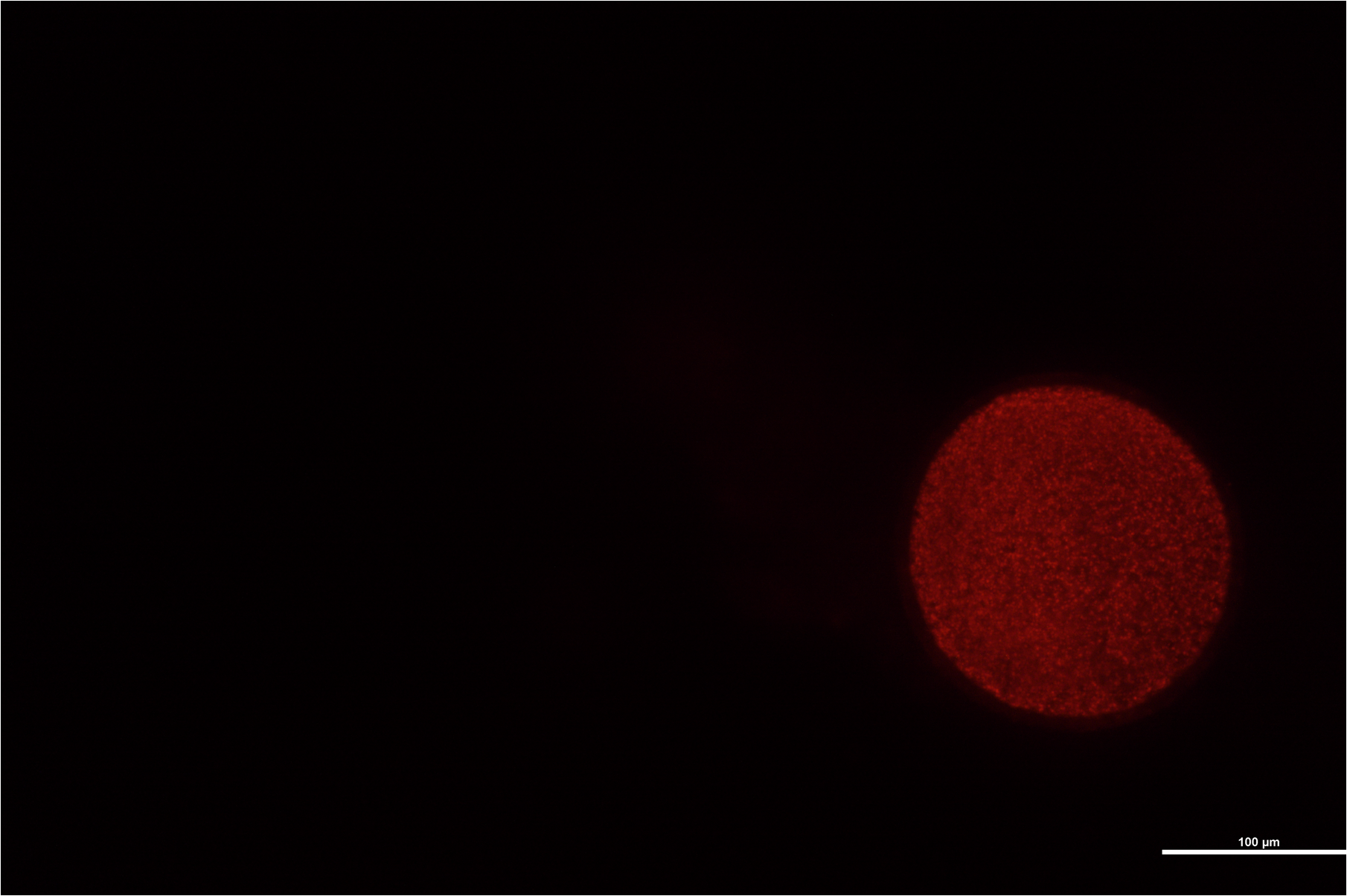

Supplement: Supplemental Information 3 — A: mitochondrial better distribution. B-E: mitochondrial heterogeneous and uneven distribution. [file peerj-08-9913-s003.zip › Aú║better distribution.tif]

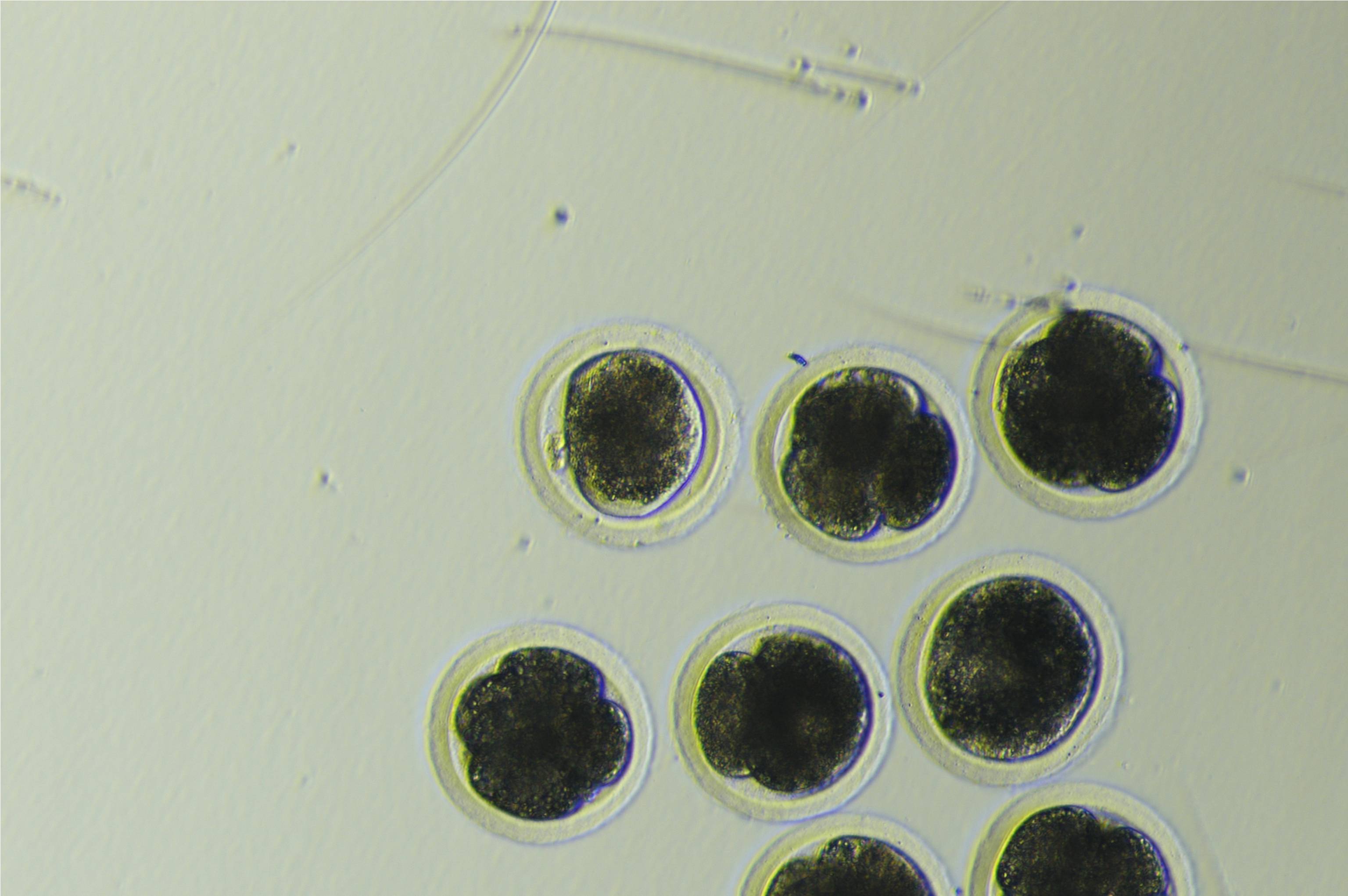

Supplement: Supplemental Information 4 — (A) cleavage. (B) blastocysts. [file peerj-08-9913-s004.zip › A, cleavage.jpg]

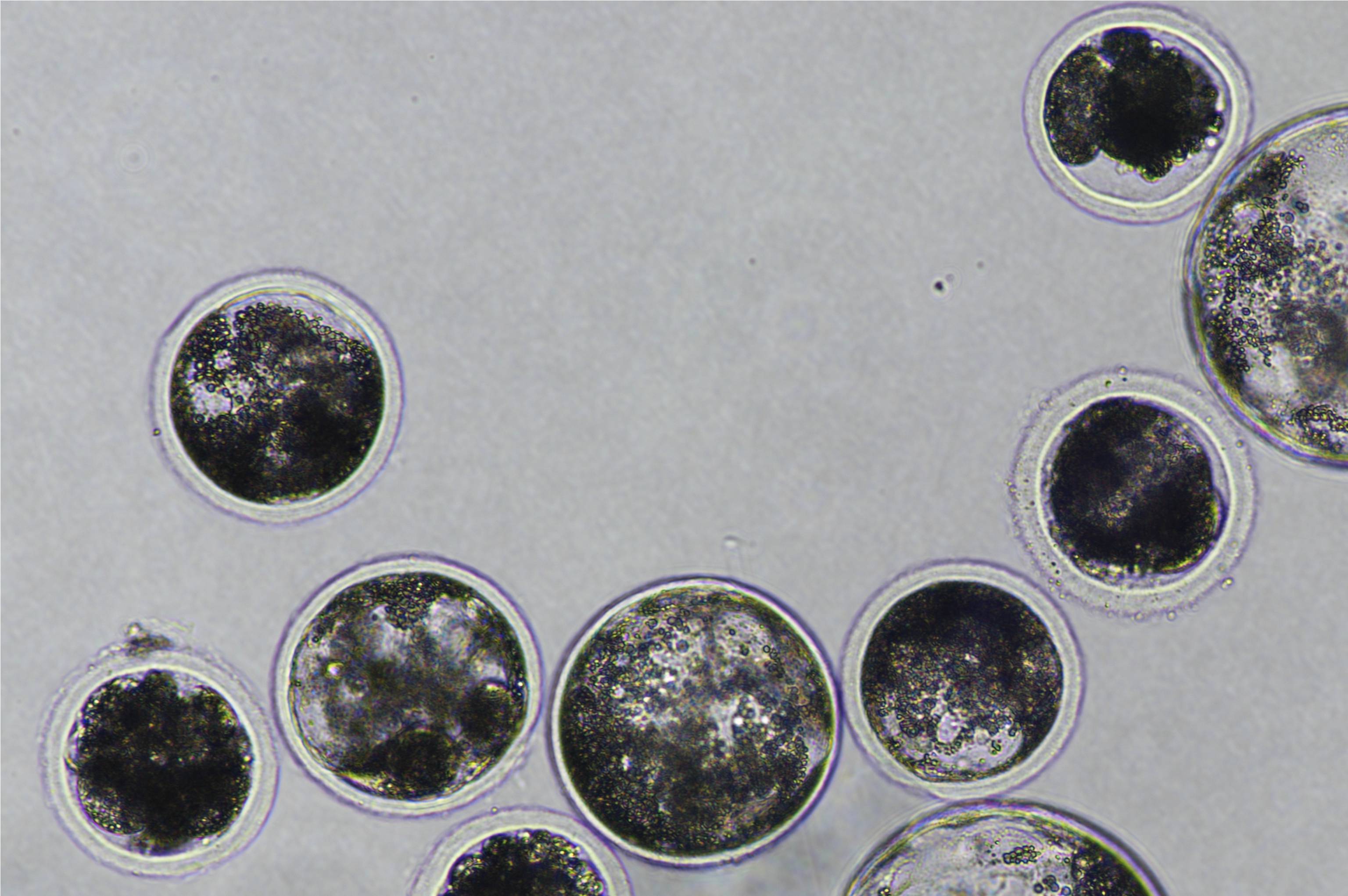

Supplement: Supplemental Information 4 — (A) cleavage. (B) blastocysts. [file peerj-08-9913-s004.zip › B, blastocyst.jpg]
